# Editorial: Anticoagulation in cardiovascular diseases: evolving role, unmet needs, and grey areas

**DOI:** 10.3389/fcvm.2023.1219033

**Published:** 2023-10-10

**Authors:** Antonino Tuttolomondo, Pasquale Pignatelli, Roberto Pola

**Affiliations:** ^1^ Department of Health Promotion, Mother and Child Care, Internal Medicine and Medical Specialties (ProMISE), University of Palermo, Palermo, Italy; ^2^ Department of Internal Medicine, La Sapienza University, Rome, Italy; ^3^ Department of Geriatric, Orthopedic, and Rheumatologic Sciences, Fondazione Policlinico Universitario A. Gemelli IRCCS, Università Cattolica del Sacro Cuore, Rome, Italy

**Keywords:** anticoagulation, thrombosis, heparin, direct oral anticoagulant (DOAC), vitamin K antagonist (VKA), atrial fibrillation, venous thromboembolism (VTE), ischemic cardiovascular disease

**Editorial on the Research Topic** Anticoagulation in cardiovascular diseases: evolving role, unmet needs and grey areas

Thrombosis is a key pathophysiological mechanism for many serious cardiovascular diseases, including deep vein thrombosis (DVT), pulmonary embolism (PE), acute coronary syndrome (ACS), and ischemic stroke.

Thrombi consist of aggregated platelets, fibrin, and trapped cells, with the distribution of these components differing substantially between arterial and venous thrombi (1). The main component of arterial thrombi is constituted by platelets. These thrombi usually originate in high-shear conditions as a result of atherosclerotic plaque disruption in arteries (2). Fibrin is instead the main component of venous thrombi. These usually arise under low-shear conditions as a result of blood stasis or hypercoagulability. As we will see, such differences may potentially influence the choice of antithrombotic therapy in individual patients. This goes along with the fact that antithrombotic therapy has evolved in a substantial manner over the last decade. Indeed, there is now a wide availability of novel medications—i.e., direct oral anticoagulants (DOACs)—and the promise of even newer anticoagulants entering the field in the very near future. In addition, novel clinical and therapeutic indications to anticoagulation have emerged, as in the case of the prevention of arterial events in subjects with ischemic cardiovascular diseases. Nonetheless, these advancements have increased the appreciation that many unmet needs and grey areas still exist when dealing with patients who need antithrombotic therapies, and this is the reason why we have decided to put together, in a dedicated Research Topic, up-to-date contributions from researchers who have personal and documented experience in the field of anticoagulant therapy.

For several decades, vitamin K antagonists (VKAs) have been the treatment of choice for long-term oral anticoagulation. Unfortunately, VKAs have many disadvantages that limit their use in the real world. Almost all the limitations of VKAs have been overcome by DOACs, which are at least as effective as VKAs in preventing thrombotic events in patients with non-valvular atrial fibrillation (NVAF) and venous thromboembolism (VTE), with the advantage of being safer in terms of bleeding risk (especially intracranial hemorrhage) and easier to use (2). This has led to a complete change in the therapeutic scenario of anticoagulant regimens. According to all the international guidelines, DOACs are now the treatment of choice for patients with NVAF and VTE. This is also valid in frail and elderly patients and in subjects with kidney insufficiency, at least for glomerular filtration rates above 30 ml per minute (3, 4). The success of DOACs has led scientists to investigate their possible use also in additional clinical settings, such as the prevention of embolic strokes of unknown source (ESUS), which represents about 25% of all ischemic strokes, and the reduction of cardiovascular events in subjects with arterial ischemic diseases. If the studies on ESUS have produced inconsistent results (5–7), those on ischemic cardiovascular diseases have led to important clinical and therapeutic advancements. The landmark example is the fact that nowadays there is a specific DOAC—i.e., rivaroxaban—that has become part of the therapeutic armamentarium of doctors who treat patients with peripheral artery disease (PAD). This is the result of the evidence provided by the COMPASS and VOYAGER trials, which have demonstrated that in patients with PAD, a dual antithrombotic therapy, consisting of the addition of a so-called vascular dose of rivaroxaban (2.5 mg twice daily) to an antiplatelet agent, reduces the risk of cardiovascular death, myocardial infarction, stroke, and limb adverse events (8, 9).

Hard to believe until a while ago, DOACs—which have been called “novel anticoagulants” for many years—might become “older” very soon. This is because strategies that target coagulation factors XI and XII (FXI and FXII) might soon hit the market. These newer medications promise to have the ability to limit thrombosis growth with an impact on hemostasis that is lower than that of DOACs (10). Very recently, a study conducted on patients with NVAF treated with an FXI inhibitor—i.e., abelacimab—was stopped early because of an overwhelming reduction in bleeding compared to a DOAC (11). A grey zone in our knowledge of anticoagulation efficacy is stroke prevention in hemodialysis patients with NVAF, owing to the fact that there is no strong demonstration that DOACs can be used in an efficacious and safe manner in this type of patient. This is one of those settings in which new inhibitors of coagulation factors might provide a great advantage. The clinical potential of FXI- and FXII-directed anticoagulant strategies will be better clarified over the next few years. Current FXI and XII inhibition strategies include antisense oligonucleotides (ASOs) that reduce the hepatic synthesis of clotting proteins, monoclonal antibodies that block the activation or activity of coagulation factors, aptamers, and small molecules that block the active site or induce allosteric modulation.

In the special issue that we have edited, the authors have tried to address some of the unmet needs in the field of anticoagulation in cardiovascular diseases. A contribution that was much appreciated, in terms of both visualizations and citations, was the review by Pastori et al. on the use of DOACs in patients with antiphospholipid syndrome (APS). This is indeed a delicate issue, with controversial recommendations among the different international guidelines. For instance, the European Society of Cardiology (ESC) recommends against the use of DOACs in APS patients. However, the authors correctly argue that these recommendations do not make any distinction between single-, double-, and triple-positive APS patients, between patients who only had venous thrombotic events and those who had arterial events, nor between different DOACs. This is despite the fact that these recommendations are exclusively based on the results of the Trial on Rivaroxaban in AntiPhospholipid Syndrome (TRAPS) (12), which had at least three main limitations: it included only triple-positive APS patients; some of the enrolled patients had not only venous but also arterial previous events; only a specific DOAC, i.e., rivaroxaban, was used. Extending the results of this trial to single/double-positive APS patients who only had venous thrombotic events might not be correct. It might also be questionable to extend to all DOACs the results of a trial that only used rivaroxaban. It is probably for these reasons that other international societies have decided that it is important to make some distinctions between the different clinical phenotypes of APS patients (13–15). According to these societies, there is the possibility of using DOACs in some specific situations. For instance, a patient who is diagnosed with APS when they are already on stable anticoagulation with a DOAC because of a previous VTE might continue DOAC treatment as the benefit of switching to VKAs may not be certain in this case. Likewise, a patient with severe INR instability while on a VKA might benefit more from a stable anticoagulation with a fixed-dose DOAC. There are also patients who are unwilling to take a VKA or unable to undergo regular INR monitoring. In these cases, DOAC treatment might be taken into consideration. Finally, there might be patients with contraindications to VKA therapy, who might therefore be considered for DOAC treatment.

The research paper by Fu et al., which compared the relative risk of embolism and major bleeding between apixaban and warfarin in patients with NVAF and compromised kidney function, was also highly viewed. The use of DOACs in subjects with severe chronic kidney disease (CKD), i.e., hemodialysis patients, was the focus of a meta-analysis by Elfar et al. This contribution is of interest because hemodialysis patients have been excluded from clinical trials on DOACs, and therefore, the evidence of their efficacy and safety is weak in this cohort.

Many contributions to our Research Topic consisted of articles on anticoagulation in frail subjects, including those with cancer, dementia, and increased risk of falling Parsi et al., Liu et al., Zeng et al., Gao et al. These articles are important because they reflect the need for physicians to better understand how to treat frail older adults in real life.

Other contributions that merit mention are the review by Gottsäter, which focused on the rationale for recommendations on dual antiplatelet and anticoagulant treatment in subjects with peripheral artery disease (PAD), the original cross-sectional study by Suo et al. on the evolution of antithrombotic treatments for patients with AF and coronary syndromes in China, and the mini-review by Hardy et al. on the possible importance of DOAC level for an uninterrupted DOAC approach for catheter ablation in AF.

In this Research Topic, there were also contributions on anticoagulation during cardiac surgery. Other contributions were from Wu et al. on the association between the use of anticoagulants and bone fractures, Mirijello et al. on pulmonary artery stump thrombosis, Prouse et al. on the possibility of using the SOFA Score to identify subjects at high risk for VTE among those affected by SARS-CoV-2, Li et al. on a nomogram to predict left atrial thrombus in patients with AF, Liu et al. on the reappraisal of DOACs in AF patients, Lin et al. on the differences in the presentation of arterial thrombotic events between patients with a history of VTE or AF, Liu et al. on intraocular bleeding in patients with AF treated with different anticoagulants, Meihandoest et al. on a heparin-calibrated anti-Xa assay, Liu et al. on the evidence available on DOACs vs. VKA in Latin American patients with AF, Liu et al. on the risk of diabetes in patients with AF treated with DOACs compared to VKA, Cao et al. on anticoagulation in AF patients who have bioprosthetic heart valves, Li ei al. on the clinical characteristics and prognosis of patients with left ventricular thrombus in China, and Gao et al. on the use of sodium alginate hydrogel coatings on extracorporeal membrane oxygenation for anticoagulation.
